# Factors Contributing to Achilles Tendon Re-rupture: A Systematic Review

**DOI:** 10.7759/cureus.99907

**Published:** 2025-12-23

**Authors:** Abimbola O Kolawole, Temidun O Kolawole, Robert H Ablove

**Affiliations:** 1 Medicine, Central Michigan University College of Medicine, Mount Pleasant, USA; 2 Medicine, Oakland University William Beaumont School of Medicine, Rochester, USA; 3 Orthopedic Surgery, Jacobs School of Medicine and Biomedical Sciences, Buffalo, USA

**Keywords:** achilles rupture, achilles tendinopathy, preventative measures, re-rupture, sports participation

## Abstract

Acute Achilles tendon rupture (ATR) is common among active adults, yet factors associated with re-rupture remain inconsistently reported. Because available studies differ substantially in design, follow-up duration, and outcome definitions, this systematic review evaluates which treatment strategies and patient-related factors are associated with Achilles tendon re-rupture without overstating precision.

Following Preferred Reporting Items for Systematic Reviews and Meta-Analyses (PRISMA) 2020 guidelines, we systematically searched PubMed, Web of Science, Cumulative Index to Nursing and Allied Health Literature (CINAHL), and the Cochrane Library (2004-October 23, 2024) for studies reporting re-rupture after acute ATR. Eligible studies included adults with clinically and/or imaging-confirmed ATR and evaluated associations between baseline characteristics, treatment approaches, or rehabilitation strategies and subsequent re-rupture. Owing to substantial heterogeneity, data were synthesized narratively, and crude numerical ranges were reported descriptively. Study quality was assessed with the National Institutes of Health (NIH) Quality Assessment Tools.

Eleven studies (44,557 patients) met the inclusion criteria. Reported re-rupture rates varied widely across treatment strategies, reflecting heterogeneous follow-up periods, definitions of re-rupture, and surgical techniques. Higher recurrence rates reported with specific techniques, such as the Tenolig percutaneous system, were derived from small, non-randomized studies and should be interpreted cautiously. More broadly, available evidence within the included studies did not consistently identify a superior standard repair method. Early weight-bearing (EWB) and functional rehabilitation, when examined, did not demonstrate a clear association with higher re-rupture frequency, though protocols varied substantially. Baseline factors such as male sex and pre-existing tendinopathy were observed more frequently among patients who sustained re-rupture, but findings were inconsistent across studies.

Re-rupture after ATR appears to occur within a multifactorial context involving patient- and treatment-related characteristics, but current evidence does not clearly favor one contemporary repair strategy over another. Early functional rehabilitation was commonly implemented without higher observed re-rupture frequencies within structured protocols. Future studies should use consistent definitions, standardized rehabilitation pathways, and more uniform outcome reporting to improve comparability.

## Introduction and background

The Achilles tendon is the strongest and largest tendon in the human body, connecting the gastrocnemius-soleus complex to the calcaneus and enabling essential functions such as walking, running, and jumping [1]. Despite its strength, it is particularly vulnerable to injury because it is subjected to high tensile loads, especially during sudden acceleration, deceleration, or changes in direction [1]. Achilles tendon rupture (ATR) is therefore a common sports-related injury [1], and its clinical significance lies not only in the immediate loss of plantarflexion strength but also in the potential for long-term functional deficits and the occurrence of re-rupture.

Participation in sporting activities has steadily increased from the 1990s through the 2000s [2], coinciding with a rising incidence of ATR [3]. Epidemiologic studies show that rupture rates increased from 2.1 per 100,000 individuals annually in 1979 to 21.5 per 100,000 individuals annually in 2011 [4]. Acute ATR occurs most frequently in men in their 30s and 40s who participate in recreational or competitive sports [5,6]. Recreational sports account for approximately 75% of ruptures, while competitive sports account for 8-20% [6].

Following primary treatment, Achilles tendon re-rupture is a major clinical concern because recurrence is frequently associated with more complex management, prolonged rehabilitation, and worse long-term outcomes [7]. One study reported a mean re-rupture rate of 0.94 per 100,000 individuals annually across all ages and 1.16 per 100,000 among adults [8]. However, the specific factors associated with re-rupture remain unclear [9].

Several potential factors have been explored in the literature. Early weight bearing has been investigated for its possible association with ATR re-rupture rates [10], while a retrospective cohort study examined relationships between gender, comorbidities, and re-rupture occurrence [11]. Sports involving rapid direction changes and high-speed footwork are known to be commonly associated with primary rupture [6], yet the relationship between sport participation and re-rupture remains uncertain [11]. Seasonal variation has also been evaluated, with recent findings suggesting a correlation between the season of initial injury and subsequent re-rupture rates [12].

ATRs can be managed using non-surgical or surgical approaches [13]. Percutaneous Tenolig repair is categorized as a percutaneous technique, whereas open surgery and minimally invasive repair represent surgical methods [14]. A recent multicenter study compared re-rupture rates among these techniques [15]. Additionally, two randomized controlled trials assessed how the timing of weight bearing after repair relates to re-rupture occurrence [16,17], and several other studies have examined repair method and corresponding outcomes [18-22]. Rehabilitation protocols generally involve either immediate or delayed weight bearing following repair.

This systematic review aims to determine which treatment strategies and patient-related factors are associated with Achilles tendon re-rupture after an acute rupture. Specifically, we evaluate how surgical versus non-surgical management, differences among surgical techniques, and variations in rehabilitation protocols relate to re-rupture rates, while also examining whether baseline characteristics, such as sex, age, and pre-existing tendinopathy, are observed more frequently among patients who experience re-rupture.

## Review

Methods

Data Sources and Search Strategy

This review followed the Preferred Reporting Items for Systematic Reviews and Meta-Analyses (PRISMA) 2020 guidelines for new systematic reviews [23]. Two reviewers independently searched four databases, PubMed, Web of Science, Cochrane Library, and CINAHL Plus with Full Text (EBSCO), to identify relevant studies published from 2004 to October 23, 2024. Search terms included Achilles tendon, rupture, re-rupture, Achilles tendon injury, and Achilles tendon treatment. The search was limited to English-language articles, and all levels of evidence were eligible for inclusion. Duplicate records and studies with abstracts not relevant to Achilles tendon re-rupture were excluded during screening. The review protocol was registered with INPLASY (INPLASY202470102).

Eligibility Criteria and Selection Process

The study selection process followed standard two-stage screening (title/abstract followed by full text), with two reviewers, AOK and TOK, working independently. Disagreements were resolved through consensus discussion, consistent with PRISMA and NIH guidance.

The inclusion criteria for articles were determined using the PICOS (Participants/Interventions/Comparisons/Outcomes/Study Design) principles. The population of interest included adults with clinically and/or imaging-confirmed acute ATR. Studies that reported patient characteristics such as sex, age, BMI, activity level, or comorbidities (e.g., tendinopathy) were eligible, although these factors were treated strictly as baseline characteristics associated with re-rupture rather than as interventions. The interventions evaluated encompassed a broad range of treatment strategies for acute ATR, including open surgical repair, minimally invasive or percutaneous repair (with Tenolig considered a subtype of percutaneous technique rather than a standalone intervention), non-operative functional rehabilitation, early weight bearing or early mobilization protocols, and adjunctive perioperative management such as immobilization methods or postoperative boot type. Comparators included alternative surgical techniques (e.g., open versus minimally invasive repair), surgical versus non-surgical treatment, early versus delayed weight bearing, and variations in postoperative immobilization or rehabilitation approaches. The outcome was the rate of Achilles tendon re-rupture following treatment. Eligible study designs included randomized controlled trials, prospective and retrospective cohort studies, case-control studies, and large registry analyses. Case series <10 patients, biomechanical studies, cadaveric studies, and non-clinical reports were excluded.

Exclusion criteria included (1) systematic reviews and meta-analyses, (2) publications for which full text was not available, and (3) studies that did not investigate factors associated with Achilles tendon re-rupture rates.

Data Extraction

One reviewer (AOK) extracted data from the articles, and the other reviewer (TOK) cross-checked the extraction for accuracy. The extraction process employed a structured, predefined template with independent verification by the second reviewer. Data collected included study sample characteristics (sex of participants and mean age), Achilles tendon re-rupture rates after follow-up, use of percutaneous Tenolig repair, open surgery, and minimally invasive surgery repair, season and sport in which ATR was sustained, and onset of weight bearing after tendon repair.

Risk of Bias

To assess the methodological quality of the studies, two independent reviewers, AOK and TOK, used the National Institutes of Health Quality Assessment Tool for Observational Cohort and Cross-Sectional Studies, Quality Assessment of Controlled Intervention Studies, and Quality Assessment Tool for Before-After (Pre-Post) Studies With No Control Group (see Appendices) [24]. These tools include 14 questions addressing internal validity, reporting biases, and potential flaws in study methods or implementation. Studies were classified as good (score ≥80%), fair (score between 79% and 50%), or poor (score <50%).

Data Synthesis

Data were synthesized by combining and comparing re-rupture rates associated with sport participation, season of the year, sex, comorbidity, repair technique, and onset of weight bearing after tendon rupture repair. These findings were summarized, analyzed, and recorded in a standardized tabular form created by the authors. A narrative synthesis was also used to summarize the evidence from the included studies. Variables such as season of injury and sport participation were included to provide contextual information regarding the circumstances of the initial rupture and were not treated as independent or causal predictors of re-rupture risk. The narrative synthesis approach was chosen intentionally because of substantial heterogeneity in interventions, definitions, follow-up periods, and outcome reporting across studies. In such circumstances, PRISMA and SWiM (Synthesis Without Meta-Analysis) recognize narrative synthesis as acceptable and preferable to meta-analysis, which could yield misleading pooled estimates. Accordingly, any numerical ranges reported in this review (e.g., rates of complications or re-rupture) are presented solely as crude, descriptive ranges extracted directly from individual studies rather than as quantitative estimates or comparative effect sizes. These values should not be interpreted as precise or generalizable rates but rather as an illustration of the variability observed across heterogeneous study designs and populations. This approach aligns with established guidance on narrative synthesis and ensures that numerical data are contextualized rather than aggregated.

Results

Study Selection 

**Figure 1:**
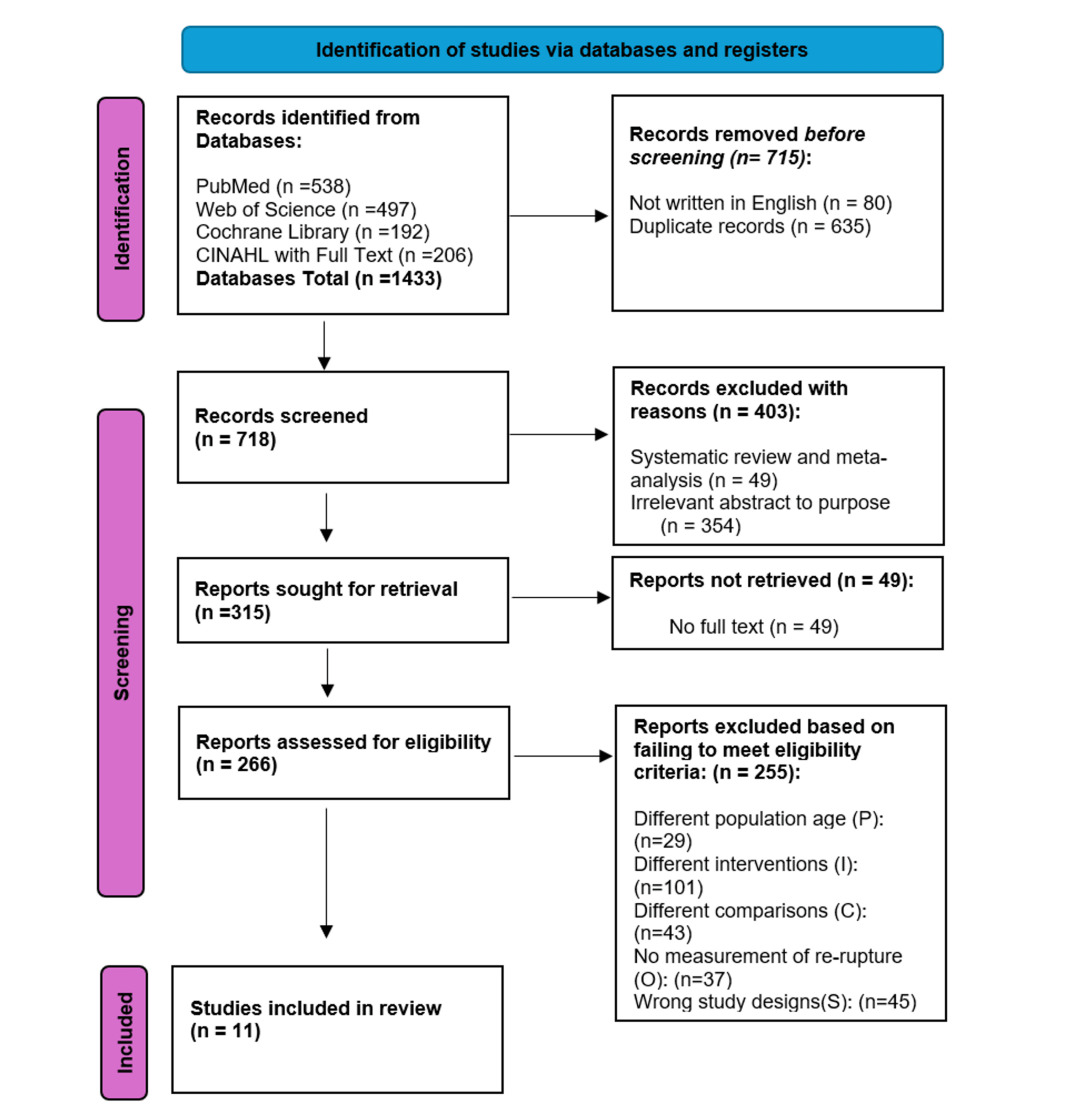
The PRISMA flow chart PRISMA: Preferred Reporting Items for Systematic Reviews and Meta-Analyses.

The results of the study selection process using a PRISMA flow diagram are shown in Figure 1. Overall, 1433 records were identified from the databases. A total of 718 records were included for screening. After screening, 315 full-text reports were sought for retrieval, and 266 final reports were accessed for eligibility; 11 studies were included in the systematic review.

Data Extraction

The characteristics of the included participants across the studies are shown in Table 1. A total of 44,557 participants were included in this systematic review across 11 studies. The mean age of all participants was 39.6 years. There were a total of 36,713 male participants, 7,552 female participants, and 292 participants for whom sex was not specified.

The first study was a retrospective study including 349 patients with unilateral acute ATR, prospectively treated with standardized surgical techniques. Participation in high-risk sports and the meteorological season at the date of injury were noted. One-year follow-up monitored Achilles tendon re-rupture rates [12]. The second study was a prospective, non-randomized study with 111 patients with acute ATR. Seventy-four patients underwent open repair surgery, 22 underwent percutaneous repair using Tenolig, and 15 underwent minimally invasive repair. Six-month follow-up monitored Achilles tendon re-rupture rates [15]. The third study was a randomized controlled trial with 47 patients with ATR treated conservatively. Twenty-three patients were treated with partial weight bearing beginning on the same day as conservative treatment, and 24 patients were treated with non-weight bearing after a four-week period. Six- and twelve-month follow-up monitored Achilles tendon re-rupture rates [16].

The fourth study was a pre-post study with no control group and included 273 patients with acute ATR. Patients were treated using ultrasound examination, functional orthoses, early weight bearing, and an accelerated exercise regimen. Achilles tendon re-rupture was assessed at four, six, and nine months [10]. The fifth study was a retrospective cohort study analyzing 43,287 patients surgically treated for ATR between 2008 and 2018. The presence of Achilles tendinopathy and sex were examined for association with re-rupture [11]. The sixth study was a randomized controlled trial involving 64 patients with ATR. Thirty-four patients were enrolled in an early weight-bearing protocol, and 34 patients were enrolled in a late weight-bearing protocol. ATR re-rupture rates were measured at three, six, and twelve months after surgery [17].

The seventh study was a prospective cohort study with 52 patients with ATR treated with percutaneous repair using resorbable sutures. Rates of re-rupture and postoperative complications were collected at one, three, six, and twelve months of follow-up [18]. The eighth study was a prospective cohort study with 292 patients with ATR. Two hundred twelve patients were treated with surgical repair and plaster immobilization for six weeks, and 80 patients underwent non-surgical repair with splinting for twelve weeks. Re-rupture rates and postoperative complications were assessed at six to twelve months or longer of follow-up [19]. The ninth study was a prospective cohort study with 30 patients who underwent percutaneous repair for ATR followed by accelerated immobilization. Re-rupture rates, postoperative function, and complications were assessed at three, six, and twelve months or longer [20].

The tenth study was a retrospective study of 15 patients with ATR treated with minimally invasive repair. Re-rupture rates, postoperative complications, and function were collected [21]. The eleventh study was a prospective study with 53 patients with ATR. Thirty patients were treated with limited open repair, and 23 were treated with percutaneous repair. Postoperative re-rupture rates, complications, and function were assessed over a nine-month or longer follow-up period [22] (Table 1).

**Table 1:** Extracted data from included studies N/A: Not available, ATR: Achilles tendon rupture, RCT: randomized controlled trial, SMART: supervised modern accelerated rehabilitation technique, WB: weight bearing, sig.: significant.

Study (Year)	Study Type	Sample (n, Mean Age, % Male)	Main Aim	Repair/Intervention Groups	Re-rupture Rate
Saarensilta et al. (2020) [12]	Retrospective cohort	349, mean 39.8 years, 77% M	Assess meteorological season & ATR outcomes	Seasonal comparison	Summer 7%, Spring 0%, Autumn 2%, Winter 1%
Laboute et al. (2023) [15]	Prospective cohort	111, mean 41.8 years, 81% M	Compare re-rupture & outcomes across surgical techniques	Open, Percutaneous, Minimally invasive	Open 1.3%, Tenolig 27%, Min. 0%
Korkmaz et al. (2015) [16]	RCT	47, mean 37 years	Compare early vs late weight bearing	Early n=24, Late n=23	Early 17.5%, Late 12.5% (p=0.81)
Hutchison et al. (2015) [10]	Pre-post (no control)	273, mean 46.5 years, 79% M	Assess SMART functional protocol	Early WB protocol	3 re-ruptures total
Choi et al. (2024) [11]	Retrospective cohort	43,287, 42.2 years, 77% M	Identify risk factors (gender, tendinopathy)	Surgical ATR	Males 82% (p<0.014), Tendinopathy 26% (p<0.001)
Deng et al. (2023) [17]	RCT	68, mean age N/A	Evaluate early vs late WB after minimally invasive repair	Early n=34, Late n=34	No sig. difference
Campillo-Recio et al. (2023) [18]	Prospective cohort	52, mean 45.96 years, 92.3% M	Percutaneous repair with resorbable sutures	Percutaneous	5.77%
van der Linden et al. (2004) [19]	Prospective cohort	292, mean 37 years	Surgical vs non-surgical	Surgical 212, Non-surgical 80	Surgical 4.7%, Non-surgical 5%
Al-Mouazzen et al. (2015) [20]	Prospective cohort	30, mean 41 years, 70% M	Percutaneous repair + early rehab	Percutaneous	0%
Chan et al. (2008) [21]	Retrospective cohort	15, mean 39 years, 93% M	Minimally invasive repair outcomes	Minimally invasive	0%
Subaşı et al. (2023) [22]	Prospective cohort	53, mean 45.1 years, 64% M	Limited open vs percutaneous	Open 30, Percutaneous 23	Percutaneous 4.4%, Open 0%

Quality Assessment

The quality of the first study [12], according to the National Institute of Health Quality Assessment Tool for Observational Cohort and Cross-Sectional Studies, was 10/13 (76.9%), indicating fair quality (see Table 1). The weaknesses of the study were that it was not blinded, it only had one follow-up, and it did not statistically adjust for the impact of confounding variables on the observed association between meteorological season and Achilles tendon re-rupture rates. It did acknowledge its sample’s confounding variables. The quality of the second study [15], according to the National Institute of Health Quality Assessment Tool for Pre-Post Studies With No Control Group, was 11/12 (91.7%), indicating good quality. The weakness of the study was that an interrupted time-series design was not implemented. The quality of the third study [16] in the review, according to the National Institute of Health Quality Assessment Tool for Controlled Intervention Studies, was 11/14 (78.6%), indicating fair quality (see Table 1). There were three weaknesses of this study. (1) The study participants and providers were not blinded to treatment group assignments. (2) Similar background treatment, namely the same conservative treatment of ATR for all 47 patients, was not avoided. (3) The sample size of 47 patients was not sufficiently large to detect a difference in the re-rupture rates between groups 1 and 2 with at least 80% power. The quality of the fourth study [10], according to the NIH Quality Assessment Tool for Before-After (Pre-Post) Studies With No Control Group, was 10/12 (83.3%), indicating good quality. The weaknesses of the study were a lack of assessor blinding and not accounting for individual-level data on group effects. The quality of the fifth study [11], according to the NIH Quality Assessment Tool for Observational Cohort Studies, was 12/12 (100%), indicating good quality. The quality of the sixth study [17], according to the NIH Quality Assessment Tool for Controlled Intervention Studies, was 12/14 (85.7%), indicating good quality. The weaknesses of this study were that the assessors were not blinded and the sample size was smaller. The quality of studies 7-11 [18-22], according to the National Institute of Health Quality Assessment Tool for Observational Cohort Studies, was 11/13 (84.6%), indicating good quality. The weaknesses of these studies were a lack of blinding of assessors to participant exposure and no statistical adjustment for confounding variables. Table 2 illustrates the quality assessment of the studies using the NIH quality assessment tool.

**Table 2:** NIH quality assessment summary Not-applicable questions are not included in score total calculations.

Study	Tool Used	Score	%	Quality	Main Weaknesses
Saarensilta et al. (2020) [12]	NIH Cohort Tool	10/13	77%	Fair	Not blinded, one follow-up, no confounder adjustment
Laboute et al. (2023) [15]	Pre-Post Tool	11/12	92%	Good	No time-series design
Korkmaz et al. (2015) [16]	Controlled Intervention	11/14	79%	Fair	No blinding, small n, same conservative care
Hutchison et al. (2015) [10]	Pre-Post Tool	10/12	83%	Good	No blinding, lacked individual-level data
Choi et al. (2024) [11]	Cohort Tool	12/12	100%	Good	—
Deng et al. (2023) [17]	Controlled Intervention	12/14	86%	Good	No assessor blinding, small sample
Campillo-Recio et al. (2023) [18]	Cohort Tool	11/13	85%	Good	No blinding, no confounder adjustment
van der Linden et al. (2004) [19]	Cohort Tool	11/13	85%	Good	No blinding, no confounder adjustment
Al-Mouazzen et al. (2015) [20]	Cohort Tool	11/13	85%	Good	No blinding, no confounder adjustment
Chan et al. (2008) [21]	Cohort Tool	11/13	85%	Good	No blinding, no confounder adjustment
Subaşı et al. (2023) [22]	Cohort Tool	11/13	85%	Good	No blinding, no confounder adjustment

Narrative Synthesis

The included studies evaluated multiple intrinsic and extrinsic factors associated with ATR and re-rupture, including seasonal variation, sports participation, repair technique, rehabilitation timing, gender distribution, and pre-existing tendinopathy. Because the studies differed substantially in surgical methods, follow-up durations, and definitions of re-rupture, all numerical values below are presented as crude descriptive ranges extracted directly from individual studies and should not be interpreted as precise or generalizable estimates.

Seasonal factors: A retrospective cohort of 349 patients reported seasonal variation in ATR incidence, with most ruptures occurring in spring (31%) and winter (32%), followed by autumn (23%) and summer (14%) [12]. One-year re-rupture frequencies ranged from 0% to 7% across seasons, but these differences did not demonstrate a consistent pattern, suggesting a seasonal association with re-rupture [12].

High-risk sports: In the same cohort, ATRs most commonly occurred during badminton (n = 73, 24%), soccer (n = 69, 22%), and floorball (n = 33, 11%) [12]. These sports involve rapid acceleration, deceleration, and directional changes that impose high eccentric loads on the tendon. These data characterize injury circumstances rather than comparative relative risks across sports.

Repair technique: Reported re-rupture frequencies varied markedly across surgical and non-surgical approaches. For example, one multicenter study of 111 patients reported crude re-rupture rates of approximately 1.13% for open repair, 27.2% for Tenolig percutaneous repair, and 0% for minimally invasive repair [15]. Additional prospective cohorts reported 5.77% re-rupture after percutaneous repair in 52 patients [18], 4.7% vs. 5% for surgical vs. non-surgical treatment in a 292-patient cohort [19], and 0% following percutaneous repair with accelerated rehabilitation in 30 patients [20]. Minimally invasive repair also showed no re-ruptures in a 15-patient retrospective cohort [21]. A 53-patient comparative cohort reported one re-rupture (4.4%) in the percutaneous group and none in the limited open group [22]. These values reflect simple ranges extracted from studies with differing follow-up durations, patient characteristics, and definitions of re-rupture and should not be interpreted as comparative estimates. Overall, contemporary minimally invasive and percutaneous techniques, particularly when combined with structured functional rehabilitation, were observed to demonstrate low re-rupture frequencies, but available evidence does not clearly identify a superior technique.

Rehabilitation and weight bearing: Early weight bearing was evaluated across several study designs. A randomized controlled trial of 47 patients found re-rupture in 17.4% of those with immediate partial weight bearing compared with 12.5% in those restricted for four weeks (p = 0.81) [16]. A pre-post implementation study of 273 patients who used early weight bearing reported three re-ruptures [10], and another randomized controlled trial of 68 patients found no significant differences in clinical outcomes between early and delayed rehabilitation [11]. Although crude rates varied across studies, the collective evidence within these designs did not indicate a clear increase in re-rupture frequency with early functional rehabilitation when implemented within structured protocols.

Gender: A large retrospective cohort of 43,287 ATR patients found that 78% were male. Among 926 patients who sustained re-rupture, 82% were male (p < 0.014) [11]. These proportions reflect a consistent pattern but do not establish precise sex-specific associations due to heterogeneity in activity exposure and reporting.

Achilles tendinopathy: Within the same cohort of 43,287 patients, 10.7% had documented Achilles tendinopathy, increasing to 26% among those who sustained re-rupture (p < 0.001) [11]. This finding demonstrates a strong association between chronic degenerative tendon changes and re-rupture occurrence.

Summary: Overall, ATR and re-rupture appear to occur within a multifactorial context involving patient characteristics, activity demands, repair technique, and rehabilitation strategy. Numerical values reported in this section represent crude ranges abstracted from individual heterogeneous studies and should not be interpreted as comparative effect estimates. Current evidence within the included studies does not clearly favor one repair strategy over another, while early functional rehabilitation was frequently implemented without higher observed re-rupture frequencies when integrated into structured protocols. Greater consistency in definitions, rehabilitation pathways, and outcome reporting is needed to improve comparability across future studies.

Discussion

This systematic review examined factors associated with ATR and re-rupture, including sports participation, sex, comorbidities, seasonality, repair technique, timing of weight bearing, and pre-existing tendinopathy. Across the included studies (44,557 participants), several patterns emerged. These findings should be interpreted as descriptive trends rather than definitive effect estimates.

Seasonal trends influenced ATR incidence but did not demonstrate a consistent association with re-rupture. High-impact sports such as badminton, soccer, and floorball were commonly reported as the precipitating activities for initial rupture, but evidence directly linking specific sports participation to re-rupture was limited. Surgical technique contributed to variability in crude re-rupture frequencies, with higher rates reported in some studies using the Tenolig percutaneous system. However, these findings were drawn primarily from small, non-randomized cohorts and should therefore be interpreted cautiously. Early weight bearing did not show a clear association with higher re-rupture frequencies across studies, though protocols varied substantially. Male sex and pre-existing Achilles tendinopathy were observed more frequently among patients who sustained re-rupture in larger cohorts, though these patterns were not uniformly observed across all studies.

Comparison With Prior Evidence

Seasonal variation in ATR incidence observed in the included literature is consistent with previous reports, such as Saarensilta et al. (2020 [12]), which noted higher rupture rates during periods of increased recreational activity. High-impact sports associated with rapid acceleration and deceleration continue to be recognized as common mechanisms of ATR (Kvist, 1994 [25]; Wertz et al., 2013 [6]). However, the current review did not find consistent evidence that seasonality or sport type independently correlates with re-rupture outcomes.

Findings related to the Tenolig technique align with previous work reporting comparatively higher crude re-rupture frequencies (Laboute et al., 2023 [15]; Idarraga et al., 2021 [26]). Still, these values originate from small observational cohorts, and prior randomized and comparative studies have not demonstrated clear superiority of open over minimally invasive or percutaneous techniques when modern repair methods are used. Campillo-Recio et al. (2023 [18]) suggest that percutaneous repair remains appropriate in selected patient groups, emphasizing the need for individualized technique selection rather than universal preference.

The literature remains mixed regarding early weight bearing. Although some studies suggest potential biomechanical benefits of early mobilization (Kauwe, 2017 [27]), others caution that premature loading may exceed early tendon tensile capacity (Korkmaz et al., 2015 [16]; Deng et al., 2024 [17]). The crude re-rupture ranges observed in this review did not indicate a clear increase in risk with early weight-bearing protocols, supporting the view that structured and gradually progressive functional rehabilitation may be safely implemented.

Male sex and pre-existing tendinopathy were recurrently associated with higher re-rupture rates in larger cohorts (Maempel et al., 2022 [8]; Järvinen et al., 2005 [1]). Proposed mechanisms include greater mechanical loading, sex-based biomechanical differences, and hormonal influences such as estrogen-mediated effects on collagen elasticity (Chiodo & Wilson, 2006 [9]). Chronic degenerative tendon changes similarly reduce structural integrity and may predispose to incomplete healing (Hess, 2010 [3]).

Explaining Heterogeneity

Substantial heterogeneity across studies, including differences in surgical technique, follow-up duration, rehabilitation protocols, activity levels, and definitions of re-rupture, limited direct comparison. These methodological differences explain much of the variability in crude numerical re-rupture rates across studies. Despite this, most included studies demonstrated moderate to high methodological quality, supporting cautious interpretation of overall trends and reinforcing the appropriateness of a narrative synthesis in place of quantitative pooling.

Clinical Implications

Findings from this review highlight the multifactorial nature of ATR re-rupture occurrence. Treatment strategies should be individualized based on patient-specific characteristics, activity level, comorbidities, and tendon condition. Preventive approaches, such as eccentric strengthening and load management in high-risk athletes, have been associated with lower primary rupture occurrence (Kvist, 1994 [25]). While open repair may be preferred in cases requiring greater mechanical stability, percutaneous and minimally invasive techniques remain viable options for appropriately selected patients. Early functional rehabilitation was commonly implemented without higher observed re-rupture frequencies when performed within structured, progressively loaded protocols. Overall, individualized management remains central to optimizing outcomes following ATR.

Strengths and limitations

This systematic review adhered to PRISMA 2020 guidelines and used a comprehensive search strategy across multiple databases, ensuring broad capture of relevant literature on ATR and re-rupture. Study quality was systematically assessed using the NIH Quality Assessment Tools, enhancing transparency and methodological rigor. By emphasizing narrative synthesis in response to substantial heterogeneity across study designs, surgical techniques, follow-up durations, and outcome definitions, this review provides an integrated overview of current evidence without overstating the precision of crude numerical ranges.

Nevertheless, several limitations warrant consideration. Most included studies were observational, restricting causal inference and increasing susceptibility to bias and confounding. Small sample sizes in some cohorts, inconsistent definitions of re-rupture, and widely variable rehabilitation protocols limited comparability across studies and reinforced the need for narrative, not quantitative, synthesis. Few studies reported long-term outcomes or used standardized patient-reported outcome measures. Despite these limitations, the review synthesizes current evidence effectively and highlights key gaps that future research should address.

Recommendation for future research 

Future studies should focus on conducting high-quality, multicenter randomized controlled trials comparing standardized surgical and non-surgical interventions for ATR. There is a need for greater consistency in reporting outcome measures, particularly regarding definitions of re-rupture, tendon healing, and functional recovery, to enable meaningful comparisons and meta-analyses. Research should also aim to establish uniform rehabilitation protocols, including the timing of weight bearing and mobilization, to determine their optimal role in recovery.

Long-term follow-up studies are essential to evaluate functional outcomes, complication rates, and patient satisfaction beyond the initial recovery period. Moreover, incorporating patient-reported outcome measures (PROMs) and cost-effectiveness analyses would enhance the clinical relevance of future research. Finally, studies should seek to identify characteristics associated with poorer outcomes, such as age, comorbidities, and activity level, to guide personalized treatment approaches and improve prognosis.

## Conclusions

This systematic review found that Achilles tendon re-rupture is associated with a combination of patient characteristics, treatment choices, and rehabilitation practices. No single repair technique consistently demonstrated clear superiority, although some approaches were reported more frequently with higher re-rupture rates in the available literature. Early functional rehabilitation was commonly implemented without higher observed re-rupture frequencies, but protocols varied widely across studies. Baseline characteristics, such as sex and underlying tendon condition, were observed more frequently among patients who experienced re-rupture, though evidence remains inconsistent.

Overall, individualized management that considers patient profile, tendon health, and functional goals is essential. Future research should use standardized definitions, uniform rehabilitation protocols, and long-term follow-up to improve comparability and support stronger evidence-based recommendations for limiting re-rupture occurrence and optimizing recovery.

## Appendices

**Table 3:** National Institutes of Health (NIH) Quality Assessment Tool for Observational Cohort and Cross-Sectional Studies [24] CD: cannot determine, NR: not reported, NA: not applicable.

Criteria	Yes	No	Other (CD, NR, NA)*
1. Was the research question or objective in this paper clearly stated?			
2. Was the study population clearly specified and defined?			
3. Was the participation rate of eligible persons at least 50%?			
4. Were all the subjects selected or recruited from the same or similar populations (including the same time period)? Were inclusion and exclusion criteria for being in the study prespecified and applied uniformly to all participants?			
5. Was a sample size justification, power description, or variance and effect estimates provided?			
6. For the analyses in this paper, were the exposure(s) of interest measured prior to the outcome(s) being measured?			
7. Was the timeframe sufficient so that one could reasonably expect to see an association between exposure and outcome if it existed?			
8. For exposures that can vary in amount or level, did the study examine different levels of the exposure as related to the outcome (e.g., categories of exposure, or exposure measured as continuous variable)?			
9. Were the exposure measures (independent variables) clearly defined, valid, reliable, and implemented consistently across all study participants?			
10. Was the exposure(s) assessed more than once over time?			
11. Were the outcome measures (dependent variables) clearly defined, valid, reliable, and implemented consistently across all study participants?			
12. Were the outcome assessors blinded to the exposure status of participants?			
13. Was loss to follow-up after baseline 20% or less?			
14. Were key potential confounding variables measured and adjusted statistically for their impact on the relationship between exposure(s) and outcome(s)?			

**Table 4:** National Institute of Health Quality Assessment of Controlled Intervention Studies [24] CD: cannot determine, NR: not reported, NA: not applicable.

Criteria	Yes	No	Other (CD, NR, NA)
1. Was the study described as randomized, a randomized trial, a randomized clinical trial, or an RCT?			
2. Was the method of randomization adequate (i.e., use of randomly generated assignment)?			
3. Was the treatment allocation concealed (so that assignments could not be predicted)?			
4. Were study participants and providers blinded to treatment group assignment?			
5. Were the people assessing the outcomes blinded to the participants' group assignments?			
6. Were the groups similar at baseline on important characteristics that could affect outcomes (e.g., demographics, risk factors, co-morbid conditions)?			
7. Was the overall drop-out rate from the study at endpoint 20% or lower of the number allocated to treatment?			
8. Was the differential drop-out rate (between treatment groups) at endpoint 15 percentage points or lower?			
9. Was there high adherence to the intervention protocols for each treatment group?			
10. Were other interventions avoided or similar in the groups (e.g., similar background treatments)?			
11. Were outcomes assessed using valid and reliable measures, implemented consistently across all study participants?			
12. Did the authors report that the sample size was sufficiently large to be able to detect a difference in the main outcome between groups with at least 80% power?			
13. Were outcomes reported or subgroups analyzed prespecified (i.e., identified before analyses were conducted)?			
14. Were all randomized participants analyzed in the group to which they were originally assigned, i.e., did they use an intention-to-treat analysis?			

**Table 5:** National Institute of Health Quality Assessment Tool for Before-After (Pre-Post) Studies With No Control Group [24] CD: cannot determine, NR: not reported, NA: not applicable.

Criteria	Yes	No	Other (CD, NR, NA)
1. Was the study question or objective clearly stated?			
2. Were eligibility/selection criteria for the study population prespecified and clearly described?			
3. Were the participants in the study representative of those who would be eligible for the test/service/intervention in the general or clinical population of interest?			
4. Were all eligible participants that met the prespecified entry criteria enrolled?			
5. Was the sample size sufficiently large to provide confidence in the findings?			
6. Was the test/service/intervention clearly described and delivered consistently across the study population?			
7. Were the outcome measures prespecified, clearly defined, valid, reliable, and assessed consistently across all study participants?			
8. Were the people assessing the outcomes blinded to the participants' exposures/interventions?			
9. Was the loss to follow-up after baseline 20% or less? Were those lost to follow-up accounted for in the analysis?			
10. Did the statistical methods examine changes in outcome measures from before to after the intervention? Were statistical tests done that provided p values for the pre-to-post changes?			
11. Were outcome measures of interest taken multiple times before the intervention and multiple times after the intervention (i.e., did they use an interrupted time-series design)?			
12. If the intervention was conducted at a group level (e.g., a whole hospital, a community, etc.) did the statistical analysis take into account the use of individual-level data to determine effects at the group level?			
